# An Abnormal Case of Diabetic Myonecrosis: A Case Report and Review of Literature

**DOI:** 10.7759/cureus.36050

**Published:** 2023-03-12

**Authors:** Henrik H Ghantarchyan, Saloni Gupta, Sarkis Arabian

**Affiliations:** 1 Internal Medicine, Arrowhead Regional Medical Center, Colton, USA; 2 Critical Care, Arrowhead Regional Medical Center, Colton, USA

**Keywords:** muscle biopsy, complications, poorly controlled diabetes, necrotizing fasciitis, diabetic myonecrosis

## Abstract

Diabetic myonecrosis, also known as diabetic muscle infarct, is a rare complication of diabetes mellitus, generally associated with poor glycemic control. It is often difficult to diagnose due to its nonspecific presentation and lack of awareness of the complication. Routine laboratory investigations often do not aid in diagnosis. Magnetic resonance imaging (MRI) may assist in diagnosis but is not routinely ordered due to cost-effectiveness and nonspecific radiologic appearance. Muscle biopsy can provide a definite diagnosis; however, it is often avoided due to its invasiveness. Treatment consists of glycemic control, rest, and analgesics for pain control. Our case describes a 42-year-old male with uncontrolled diabetes who presented with four weeks of progressively worsening right-sided lower extremity pain. The patient was taken to the operating room for concern for necrotizing fasciitis; however, it was ultimately ruled out. A diagnosis of diabetic myonecrosis was made. Recommendations were given for strict blood sugar control and to start aspirin 81 mg daily. The patient was later seen in the outpatient clinic with improvement in the lower extremity pain.

## Introduction

Diabetes mellitus (DM) is commonly encountered by healthcare providers and is caused by either the insufficiency or absence of insulin. It is due to an insufficient or absence of insulin secretions or a combination of these two [1]. According to the International Diabetes Federation, there are an estimated 415 million people diagnosed with diabetes in the year 2015 worldwide. By the year 2040, this number is expected to surpass 640 million people [2].

Poor control of diabetes can result in an extensive list of microvascular or macrovascular complications [2]. A challenging microvascular complication of DM that is rarely encountered is diabetic myonecrosis (DMN), initially described in 1965 by Angervall and Sterner [3]. There is a common association of diabetic myonecrosis with diabetic nephropathy and retinopathy, 71% and 57%, respectively [4]. DMN most commonly affects the quadriceps (60-65%), hip adductors (13%), hamstrings (8%), and hip flexors (2%) [4]. A study was done by Radcliffe et al. of 43 patients with myositis reflected a predominance of DMN in males at 66% and females at 34%. The most common presenting symptoms were muscle pain and fever [5].

The pathophysiology of diabetic myonecrosis is not clear; however, various theories have been suggested, including atherosclerosis, diabetic microangiopathy, vasculitis, ischemia-reperfusion injury, and hypercoagulable states [5]. On pathology, the cells are devoid of a nucleus interstitial infiltrate composed of acute and chronic inflammatory cells. Nevertheless, diabetic renal disease is thought to be the most common risk factor for DMN [5]. As its presentation can be nonspecific, the importance of increased awareness of this disease can help distinguish diabetic myonecrosis from many other disease states with similar presentations.

## Case presentation

A 42-year-old Spanish-speaking male with a past medical history of chronic alcoholism and poorly controlled diabetes mellitus presented to the hospital for progressive right lower extremity pain lasting four weeks. The pain is described to be a sharp, severe pain localized to his right anteromedial proximal thigh. He stated it had been progressively worsening, which prompted him to visit the emergency department (ED). The pain alleviated with rest and pain medications and worsened with activity. The patient was unable to recall any inciting events. He denied any trauma to the site or insect or animal bites. He denies the use of medications at home, including statins or intravenous medications, including intravenous (IV) drugs or steroids.

He admitted to frequent improper care of himself, referring to his alcohol abuse history. The patient had been drinking approximately six beers a day for the past ten years. Additionally, he was non-adherent to his medications. He reported an approximate ten-pound unintentional weight loss for the last month. On physical exam of the right thigh, there was noted tenderness at the right anteromedial thigh with mild induration and mild warmth. There was no noted redness or rash on the right thight. The right lower extremity strength is a 3/5, limited due to pain.

On admission, the patient was hemodynamically stable and afebrile. There was no immediate concern for infection as he was afebrile, and his white blood cell count was 6.9 10^3/uL (4.5 - 11.1 10^3/uL). He was found to have a hemoglobin A1c (HbA1c) of 16.7% and an elevated end sedimentation rate (ESR) and C-reactive protein (CRP), with a normal creatinine kinase (CK) level, as seen in Table 1.

**Table 1:** Significant lab results on presentation μL = microliter, g = gram, dL = deciliter, mEq = milliequivalent, mmol = millimole, mg = milligram, mL = milliliter

Blood test results	Patient value	Reference range
Hemoglobin A1C (%)	16.7	4.3 - 6.1
Glucose (mg / dL)	558	70 - 99
Creatinine kinase (U / L)	164	30 - 170
C-reactive protein (mg / dL)	4.53	<0.50
End sedimentation rate	>140	0 - 10

**Figure 1:**
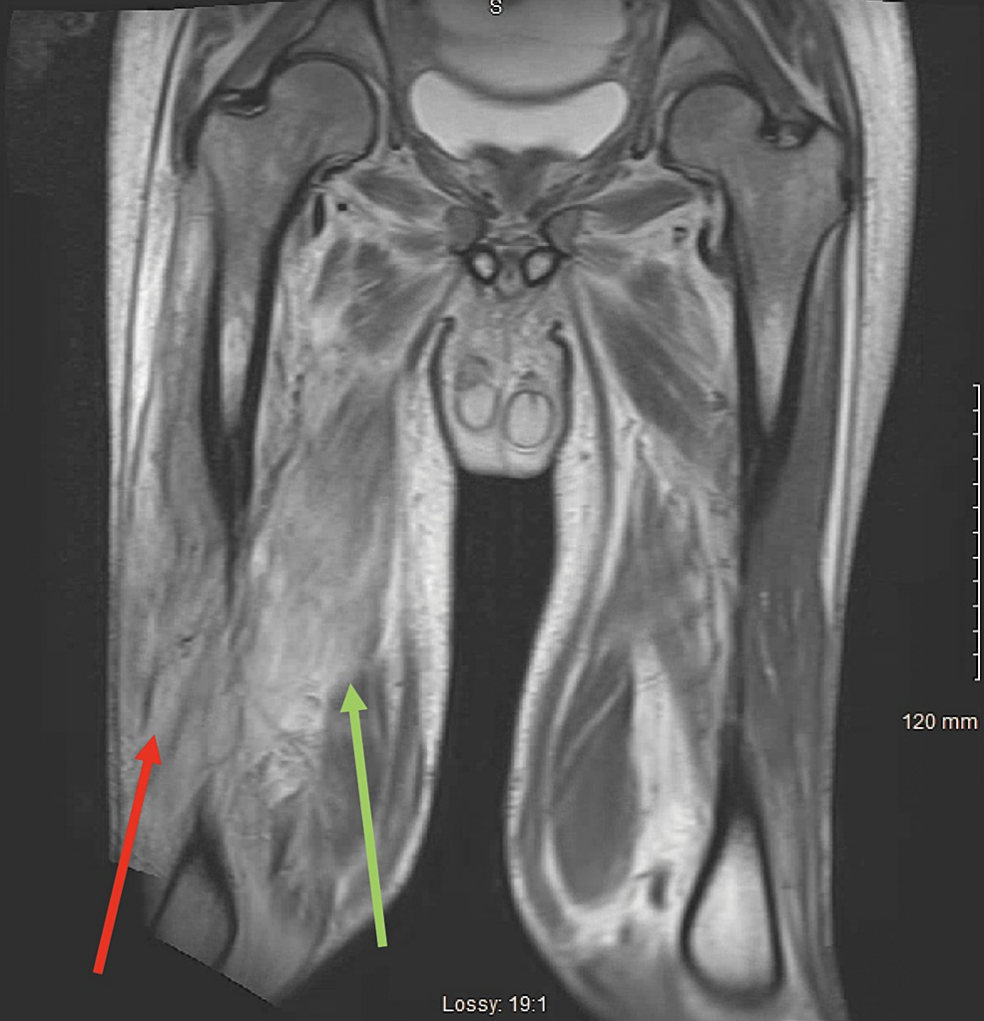
MRI of the right hip with and without contrast. T-2 weighted coronal MRI of the femur showing evidence of muscle edema and muscle enhancement involving the anterior compartment and the abductor muscles, suspicious for myositis. There are irregular areas of non-enhancement within the vastus lateralis (red arrow) and vastus medialis (green arrow) muscles which may represent myonecrosis or developing abscess.

**Figure 2:**
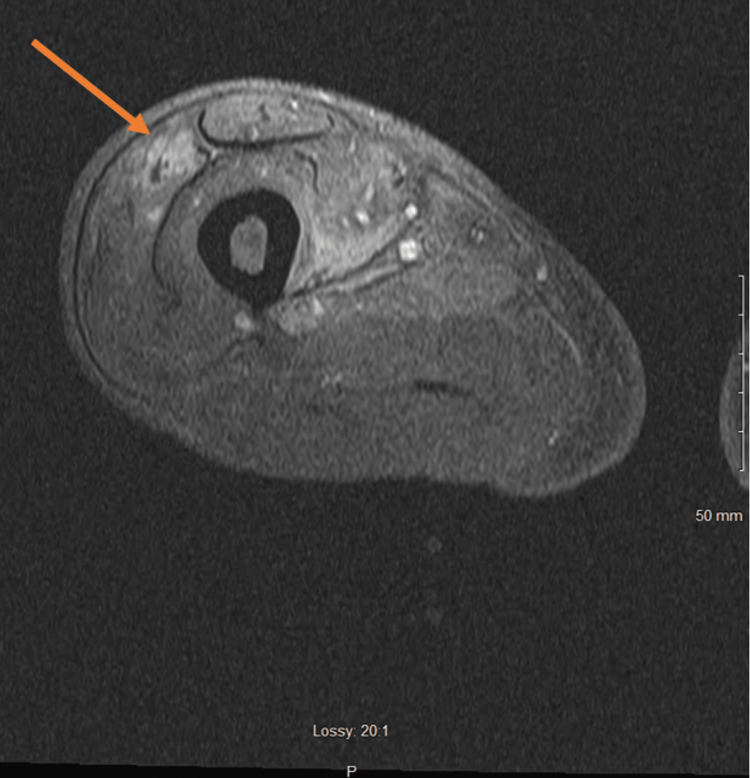
Magnetic resonance imaging of the right femur with and without contrast T-2 weighted MRI highlighting muscle edema and muscle enhancement involving the anterior compartment and the abductor muscles, suspicious for myositis. There are irregular areas of non-enhancement within the vastus lateralis and medialis muscles which may represent myonecrosis or developing abscess.

Given the patient's significant alcohol abuse history, an ultrasound of his abdomen was completed, showing ​​hepatic cirrhosis with splenomegaly and moderate ascites. A paracentesis was performed, which tested negative for spontaneous bacterial peritonitis (SBP). Bilateral lower extremity ultrasound negative for a deep vein thrombosis (DVT). An X-ray image of the right femur and hip was negative for any fractures or dislocations. Psoas abscess was ruled out using computed tomography (CT) lumbar spine and abdomen/pelvis. As the patient complained of persistent pain, magnetic resonance imaging (MRI) was obtained, which revealed severe myositis (Figures 1, 2). 

Although the patient did not display active signs of infection, such as fevers or hemodynamic instability, there was a concern for developing an abscess and even necrotizing fasciitis, given persistent thigh pain and MRI findings. The patient was started on vancomycin, dosed by a pharmacy every 24 hours, cefepime 1 gram every eight hours, and Flagyl 500 mg every eight hours. The patient was urgently taken to the operating room (OR); however, interestingly enough, there were no signs of necrotizing fasciitis, infarcted muscle, or abscess noted in the OR. All antibiotics were stopped due to this and pain was treated with aspirin 81 mg daily, hydrocodone-acetaminophen 5 mg every six hours as needed, and Toradol 30 mg IV as needed. He was also continued on strict blood glycemic control with glargine 20 units at night and lispro 5 units subcutaneously three times a day before meals along with a sliding scale as needed with adequate glycemic control. Over the remaining course of stay, he worked with physical therapy for strengthening exercises and was deemed stable for discharge one week later to his family. At the time of discharge, the patient was given extensive diabetic education and advised to continue physical therapy, analgesics as needed, and strict glycemic control. He then followed up in our outpatient clinic for closer glycemic monitoring and continued diabetic education.

## Discussion

The underlying pathophysiology of DMN is not completely understood at this time. However, a common belief is that stenosis of intramuscular vessels occurs, leading to muscle infarction. Others have proposed that diabetic microangiopathy, in combination with hypoxia-reperfusion injury, results in a severe inflammatory response, edema, and reperfusion [6]. Another proposed theory of DMN is that there is a hyper-coagulable state associated with antithrombin II deficiency, increased factor VII, hyperhomocysteinemia, presence of antiphospholipid antibodies, and decreased prostacyclin and tissue plasminogen activator levels leading to muscle infarction [6].

The importance of DMN recognition and early diagnosis lies in that it is currently underdiagnosed and difficult to recognize. Since it is a chronic disease, studies have shown that there is a high recurrence and high mortality rate. The reported recurrence rate is 34.9 to 47.8%, with the contralateral limb affected within six months, and the mortality rate is five years and is associated with the secondary complications of diabetes mellitus [3, 7]. 

As mentioned above, DMN is commonly seen in patients with poorly controlled diabetes mellitus, which was evident in our patient in the setting of an HbA1c of 16.7, with acute onset of painful swelling over four weeks prior to presentation to the ED. Commonly affected sites include the anterior thigh, which our patient had, in addition to associated mild swelling and tenderness to the site [3]. He was found to have laboratory findings of elevation in ESR (>140) and CRP (4.53), with normal CK levels (164) and no obvious leukocytosis as his white blood cell count was 6.9 10^3/uL (4.5 - 11.1 10^3/uL). MRI findings were suggestive of increased intensity of the anterior compartment and non-enhancement within the involved muscle, with concern for myositis or abscess formation.

Having a broad differential diagnosis is very important in a case as such. Some considerations include pyomyositis, abscess formation, and autoimmune idiopathic inflammatory myopathies such as dermatomyositis, polymyositis, or even necrotizing myositis. Pyomyositis is a diagnosis that we considered; however, it was less likely in our case since the patient was afebrile, had no leukocytosis, and no obvious well-defined intramuscular fluid collection suggesting bacterial muscle infection [5]. An abscess was also considered, given the MRI findings. However, the patient did not have any obvious signs of infection, as evidenced by a lack of leukocytosis, stable vital signs, and no signs of fever; furthermore, an abscess or necrotic muscle was not seen on gross observation in the OR [3]. There were laboratory findings indicating systemic inflammation; however, this is possible in cases of abscess formation or diabetic myonecrosis. 

The patient's presentation was inconsistent with dermatomyositis, polymyositis, or necrotizing myositis from autoimmune etiology, given the lack of symmetric progressive proximal muscle weakness [8]. These diagnoses do not usually present with muscle pain. The patient did not have any obvious symmetric muscle weakness in the upper or lower extremities or any obvious tenderness in different muscle areas suggestive of polymyositis. Furthermore, he had normal CK. Additionally, he did not have any obvious rashes suggestive of dermatomyositis, and no obvious heliotrope rash or gottron papules over the dorsum of fingers or knuckles was observed. There was no observed shawl sign, V-sign, or periungual erythema. He did not have any dyspnea suggestive of pulmonary findings of dermatomyositis or any chest pain suggestive of myocarditis which can be seen in dermatomyositis. Dermatomyositis can also be a paraneoplastic effect of small-cell lung cancer; however, the patient did not endorse any significant smoking history, and there was no obvious concern for lung cancer at the time [8]. 

When reviewing MRI studies of affected areas, the lack of enhancement of affected muscle after intravenous (IV) contrast administration is highly suggestive of myonecrosis. However, subacute myonecrosis may have evidence of rim enhancement, which can be mistaken for an abscess [9]. In our patient's case, both Figures 1 and 2 demonstrate irregular areas of non-enhancement within the vastus lateralis and medialis muscles, suggestive of myonecrosis or a developing abscess. This was discussed with the general surgery team in depth. Initially, there were fewer concerns for infection as our patient did not have elevated white blood cell count or systemic signs of infection like fever or tachycardia. However, given the patient's persistent pain despite one week of analgesic treatment and MRI findings concerning for underlying abscess or necrotizing fasciitis, the patient was taken to the OR for incision and drainage. No abscess or necrotizing fasciitis was discovered. The muscle was healthy, and no gross signs of necrosis were seen. Although a muscle biopsy is recommended in settings when a diagnosis of DMN is unclear, and the patient is not improving with conservative treatment [3, 7, 10], a biopsy was not obtained in this case. Generally, muscle biopsies are avoided due to prolonged wound healing and disease progression, in addition to complications such as hematomas and superimposed infections. In these cases of DMN, abscesses, infections, and even necrotizing fasciitis must be included in the differentials to prevent delay in care and serious complications [10]. 

When considering the treatment options for diabetic myonecrosis it is mainly limited to symptom management and strict glycemic control. There have been previous case reports of diabetic myonecrosis treated with low doses of anti-platelet therapy, such as oral baby aspirin. Muscle biopsy is not required for the diagnosis of diabetic myonecrosis; however, it may be considered if infection or an abscess is suspected or if the patient has an atypical presentation [3]. Muscle biopsy will demonstrate nonhemorrhagic and pale muscular tissue. Light microscopy may show areas of muscle necrosis and edema during the early stages of DMN; however, with progression to later stages, necrotic tissue will be replaced by fibrous tissue, lymphocytic infiltration, and even muscular regeneration [3]. 

Limitations

The limitations of this study are that tissue samples or cultures were not obtained when the patient was taken to the OR. Pathological evidence of microinfarctions would have aided in confirming the diagnosis of diabetic myonecrosis. Upon gross examination in the OR, there was no necrotic tissue seen. Even though necrotic tissue was not observed, this does not exclude diabetic myonecrosis as a possible diagnosis. 

## Conclusions

In conclusion, diabetic myonecrosis is a rare complication of longstanding, poorly controlled diabetes. Patients presenting with acute onset, non-traumatic muscular pain without significant relief with analgesics should be further worked up for diabetic myonecrosis as a possible differential. There are no specific markers of this disease; however, lab findings of elevated ESR, CRP, and CK are more suggestive of this differential. Imaging is an important tool, with MRI being the choice of imaging. Classical MRI findings consist of increased signal intensity in the affected intramuscular and surrounding subcutaneous tissue, as well as inflammatory changes and edema. Muscle biopsy, although not routinely indicated, can be done in cases with an atypical presentation where the diagnosis is unclear or in those where patients do not have relief even after weeks to months of analgesic treatment. Treatment is mainly conservative, with strict glycemic control and analgesics. Long-term prognosis remains poor due to underlying uncontrolled diabetes and high recurrence rates. 
